# Enhanced ethanol formation by *Clostridium thermocellum* via pyruvate decarboxylase

**DOI:** 10.1186/s12934-017-0783-9

**Published:** 2017-10-04

**Authors:** Liang Tian, Skyler J. Perot, Shuen Hon, Jilai Zhou, Xiaoyu Liang, Jason T. Bouvier, Adam M. Guss, Daniel G. Olson, Lee R. Lynd

**Affiliations:** 1 Thayer School of Engineering, Dartmouth College, 14 Engineering Drive, Hanover, NH 03755 USA; 2 Dartmouth College, Hanover, NH 03755 USA; 3 Bioenergy Science Center, Oak Ridge National Laboratory, Oak Ridge, TN 37831 USA; 4 Biosciences Division, Oak Ridge National Laboratory, Oak Ridge, TN 37831 USA

**Keywords:** Consolidated bioprocessing, *Clostridium thermocellum*, Cellulosic ethanol, Pyruvate decarboxylase, Alcohol dehydrogenase

## Abstract

**Background:**

Pyruvate decarboxylase (PDC) is a well-known pathway for ethanol production, but has not been demonstrated for high titer ethanol production at temperatures above 50 °C.

**Result:**

Here we examined the thermostability of eight PDCs. The purified bacterial enzymes retained 20% of activity after incubation for 30 min at 55 °C. Expression of these PDC genes, except the one from *Zymomonas mobilis*, improved ethanol production by *Clostridium thermocellum*. Ethanol production was further improved by expression of the heterologous alcohol dehydrogenase gene *adhA* from *Thermoanaerobacterium saccharolyticum*.

**Conclusion:**

The best PDC enzyme was from *Acetobactor pasteurianus*. A strain of *C. thermocellum* expressing the *pdc* gene from *A. pasteurianus* and the *adhA* gene from *T. saccharolyticum* was able to produce 21.3 g/L ethanol from 60 g/L cellulose, which is 70% of the theoretical maximum yield.

**Electronic supplementary material:**

The online version of this article (doi:10.1186/s12934-017-0783-9) contains supplementary material, which is available to authorized users.

## Background

Plant lignocellulosic biomass represents the most abundant renewable resource on the earth which is produced at an approximate rate of 150–170 × 10^9^ tons annually [1]. It is therefore one of the most attractive substrates for sustainable production of second-generation biofuels and organic chemicals. Among all the processes to overcome the recalcitrance of cellulosic biomass, consolidated bioprocessing (CBP) is the most promising configuration because it accomplishes biomass solubilization and fermentation in one step without adding enzymes [2–4]. *Clostridium thermocellum* is a good candidate organism for CBP due to its ability to rapidly ferment cellulose and produce ethanol. Several approaches have been pursued aimed at engineering *C. thermocellum* to produce ethanol at higher yield [5] including by-product elimination [6–9], adaptive evolution [10] and gene overexpression [11–14]; however, CBP for ethanol production from cellulose using *C. thermocellum* is still not an economical process according to the target performance metrics for cost-effective production of ethanol from lignocellulose of 90% of theoretical yield and 40 g/L titer [15].

**Fig. 1:**
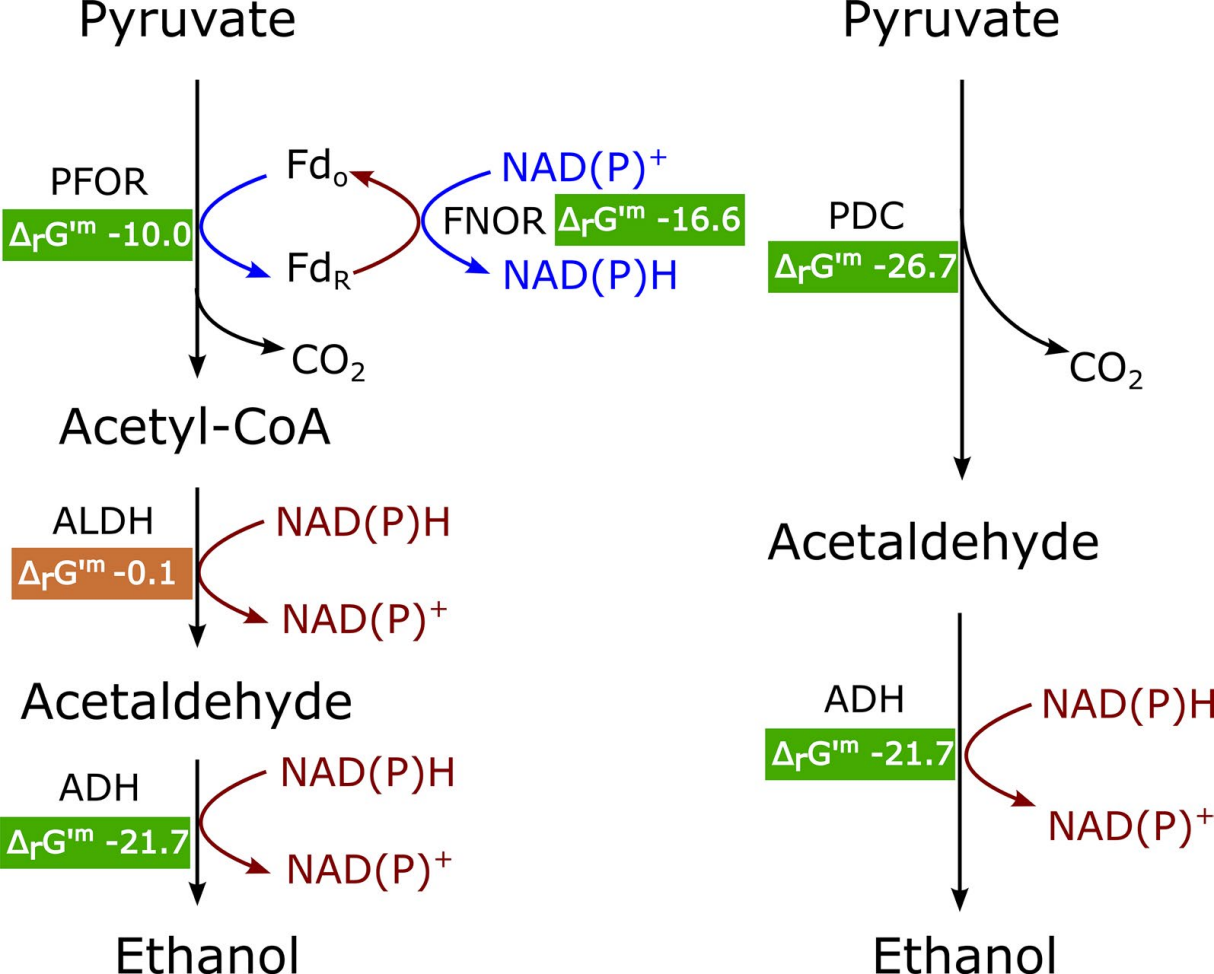
Two ethanol production pathways from pyruvate. *PFOR* pyruvate ferredoxin oxidoreductase, *ALDH* acetaldehyde dehydrogenase, *ADH* alcohol dehydrogenase, *PDC* pyruvate decarboxylase, *FNOR* ferredoxin-NAD(P)^+^ oxidoreductase, *Fdo* oxidized ferredoxin, *FdR* reduced ferredoxin. All the ΔrG′^m^ values were calculated by eQuilibrator [51] and are in units of kJ/mol. ‘m’ to mark the 1 mM concentration that is used for all reactants. For purposes of calculation, CO_2_ is assumed to be in the gas phase

One of the limiting steps for the ethanol production in *C. thermocellum* is the pyruvate ferredoxin oxidoreductase (PFOR, Fig. 1). The PFOR enzyme transfers electrons from pyruvate to ferredoxin, which must be subsequently transferred to a nicotinamide cofactor (NAD^+^ or NADP^+^) in order to be used for ethanol production. Electron transfer from ferredoxin to NAD(P) + is an exergonic reaction which is frequently coupled to another endergonic reaction for energy conservation [16]. This energy conservation, however, comes at the price of thermodynamic driving force [17]. Another potential problem with the PFOR-based pathway is its reliance on the ALDH reaction. The ΔrG′^m^ value of this reaction is − 0.1 kJ/mol (Fig. 1) and would thus require either a high ratio of acetyl-CoA to acetaldehyde or NAD(P)H to NAD(P) (or both) in order to proceed towards ethanol production.

An alternative pathway for ethanol production involves direct conversion of pyruvate to acetaldehyde by the enzyme pyruvate decarboxylase (PDC). This pathway is simpler than the PFOR pathway because it requires fewer enzymatic reactions (two vs. four for the PFOR pathway Fig. 1). Furthermore, both steps of the PDC pathway are thermodynamically favorable at standard conditions. Similar to the native pathway, the PDC pathway is also redox balanced. This pathway has been widely studied in *Saccharomyces cerevisiae* [18] and *Zymomonas mobilis* [19], but is found in a range of other organisms including *Acetobacter pasteurianus* (note that the PDC pathway in *A. pasteurianus* can also be used to produce acetate) [20], *Kluyveromyces marxianus* [21], *Zymobacter palmae* [22], *Ogataea polymorpha* [23, 24] and *Gluconobacter oxydans* [25].

The pyruvate decarboxylase gene from *Z. mobilis* has been used to enhance ethanol production in a variety of mesophilic organism including *Escherichia coli* [26], *Klebsiella oxytoca* [27], *Bacillus subtilis* [28], *Lactobacillus casei* [29] and cyanobacteria *Synechococcus* sp. [30]. When the gene was overexpressed in *Clostridium cellulolyticum*, a mesophilic cellulolytic clostridia, cellulose consumption was improved but the ethanol yield did not increase [31].

There are a number of thermophilic species of yeast that produce ethanol using the PDC pathway, but the upper temperature limit for ethanol production in these organisms appears to be around 45–48 °C [23, 24]. Although the Pdc proteins from a variety of organisms are tolerant to incubation at temperatures up to 65 °C [22], attempts to express the *pdc* gene in thermophilic organisms have met with limited success. Expression of the *Gluconobacter oxydans pdc* gene in *Geobacillus thermoglucosidasius* resulted in an increase in ethanol yield from 49% of theoretical (0.25 g ethanol/g glucose) to 69% of theoretical (0.35 g ethanol/g glucose), but this effect was only observed during growth at 45 °C and disappeared when the temperature was raised to 52 °C [32]. Kannuchamy et al. recently reported expression of the *Z. mobilis pdc* gene in *C. thermocellum* which increased ethanol titer from 1.5 g/L (26% of theoretical yield) to 3.0 g/L (53% of theoretical yield), however these yield values are significantly lower than the highest reported ethanol yields for *C. thermocellum* of 75% [10].

Here, we tested the thermostability of eight pyruvate decarboxylase candidates in vitro and then overexpressed them in *C. thermocellum*, with the goal of identifying the best candidate for ethanol production.

## Results and discussion

### Biochemical characterization of the PDC candidates

Eight thermostable pyruvate decarboxylase candidates were selected based on previous reports of thermostability (Table 1).

**Table 1:** Published data on the temperature stability of PDC from different organisms

Host	Name	T optimum (°C)	Temperature stability	Refs
*Saccharomyces cerevisiae*	*Sc*Pdc	30–45	T_1/2_^a^ is 5 min at 52.6 ± 0.4 °C	[33]
	*Sc*PdcM	40–55	T_1/2_ is 5 min at 61.8 ± 0.1 °C	
*Kluyveromyces marxianus*	*Km*Pdc	30	No data on thermostability available. The organism can grow and produce ethanol at 45–48 °C	[34]
*Zymobacter palmae*	*Zp*Pdc	55	T_1/2_ is 10 h at 50 °C and 0.4 h at 60 °C	[35]
*Ogataea polymorpha* ATCC 14754	*Op*Pdc	–	No data on thermostability available. The organism can grow and produce ethanol at 45–48 °C	[24]
*Zymomonas mobilis*	*Zm*Pdc	60	T_1/2_ is 24 h at 50 °C	[36]
*Gluconobacter oxydans*	*Go*Pdc	53	40% enzyme activity remains after 30 min at 65 °C	[32]
*Acetobacter pasteurianus*	*Ap*Pdc	65	T_1/2_ is 12 h at 50 °C and 2 h at 60 °C	[35]

^a^Half-life time

**Fig. 2:**
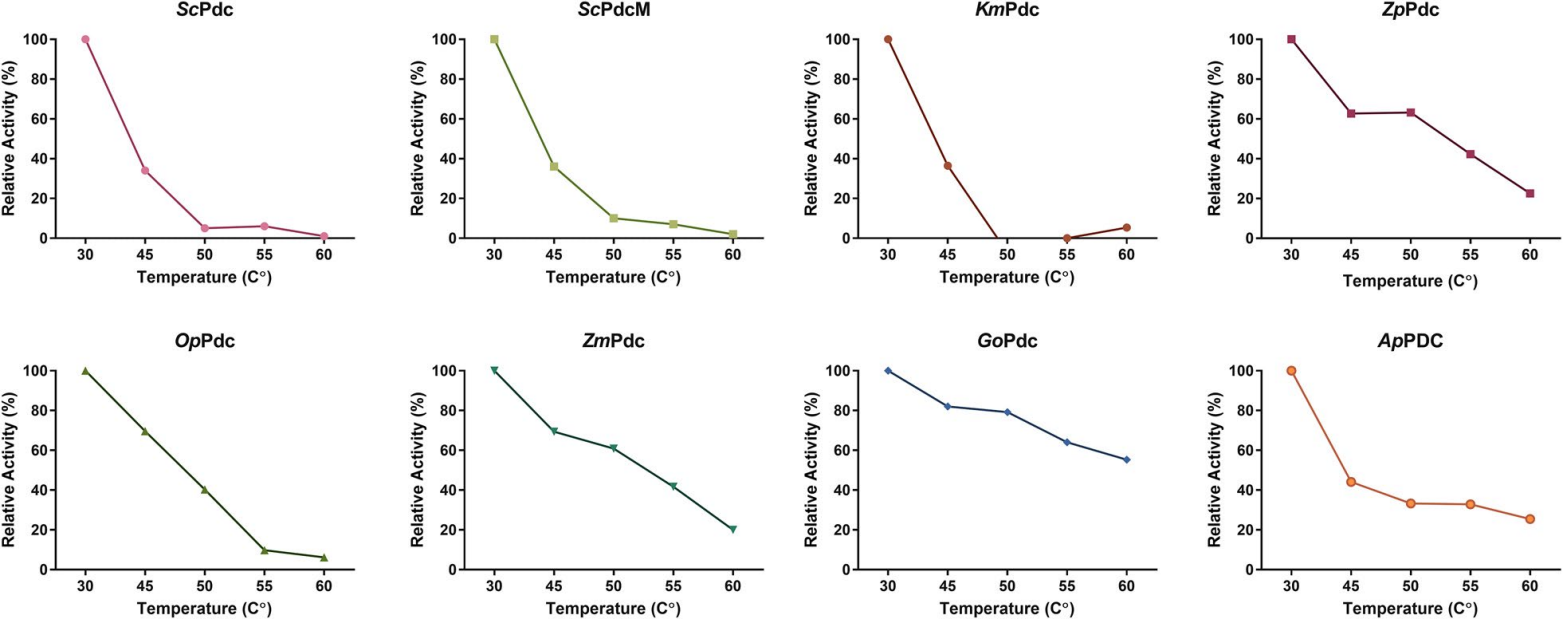
Thermostability of eight pyruvate decarboxylase candidates. Enzyme activity at 30 °C is set to 100%. Thermostability was tested at the temperatures of 45, 50, 55 and 60 °C. The data represents the average of three individual rounds of protein purification and assay. Error bars are smaller than the size of the data point markers and are therefore not shown

For our measurements of the thermostability of PDC enzymes, we used the method of Raj et al. [22] which is one of the more widely-used methods for this purpose. Candidate genes were cloned and expressed in *E. coli*. The resulting purified proteins were incubated at different temperatures from 30 to 60 °C for 30 min and then chilled on ice for 10 min before the assay. PDC activity was determined at 30 °C (Additional file 1). Relative activity was determined using the activity at 30 °C as the basis (Fig. 2). Among all eight of the candidates, only four (*Zp*Pdc, *Zm*Pdc, *Go*Pdc and *Ap*Pdc) still had more than 30% of activity after 30 min incubation at 55 °C, the growth temperature for *C. thermocellum*. Organism name abbreviations are described in Table 1.

### Heterologous expression of *pdc* genes in *C. thermocellum*

**Fig. 3:**
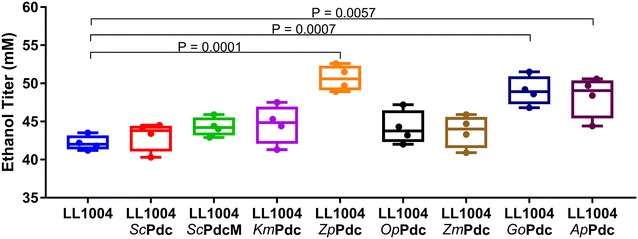
The ethanol titer of *C. thermocellum* with different pyruvate decarboxylase candidates. Four colonies were selected for each candidate *pdc* gene and evaluated by batch fermentation in MTC-5 medium with 10 g/L cellobiose. Data for each colony is represented by a single point. 10 μg/mL thiamphenicol was added to maintain the plasmid. Strain LL1004 is wild type *C. thermocellum.* The box includes the 25th to 75th percentile of data; whiskers represent 1.5 times the interquartile range

Plasmid pDGO143 was used to express all of the candidate *pdc* genes under control of the native promotor P_2638_ from *C. thermocellum* and their activities from cell extracts were measured (Additional file 2) [13]. After transformation into *C. thermocellum*, four colonies were selected for each candidate *pdc* gene, and analyzed by batch fermentation in MTC-5 medium with 10 g/L cellobiose. Three candidates, *Zp*Pdc, *Go*Pdc and *Ap*Pdc showed an increase in ethanol titer of 20, 16 and 14% respectively. For the others, the effect was less than 5% (Fig. 3). This result was consistent with the in vitro thermostability result (Fig. 2). Generally, proteins that showed high thermostability also showed increased ethanol production. The one exception was *Zm*Pdc, which exhibited high levels of thermostability in vitro, but did not increase ethanol production in *C. thermocellum*.

Our results conflict with the results of Kannuchamy et al. [37], who reported that Pdc from *Z. mobilis* improves ethanol production in *C. thermocellum*. We do not have a good explanation for this discrepancy. We tried to obtain strain and/or plasmid stocks from the authors of that work, but were told that they no longer exist and were thus not able to perform a more detailed side-by-side comparison.

In *C. thermocellum*, the bifunctional enzyme AdhE is primarily responsible for both the ADH and ALDH reactions (Fig. 1) in *C. thermocellum* [38, 39]. It has been theorized that this bifunctional enzyme can channel acetaldehyde between the ALDH and ADH domains, which limits the leakage of acetaldehyde, which is volatile and toxic [40, 41]. This channeling may reduce the ability of the ADH domain of AdhE to accept free acetaldehyde as a substrate, and could explain the high K_M_ values that have been measured for the ADH reaction in AdhE [39]. Since the product of the pyruvate decarboxylase reaction is acetaldehyde, we decided to express a dedicated ADH enzyme. Furthermore, it has been shown that AdhA from *Thermoanaerobacterium saccharolyticum* is able to increase ethanol yield in *C. thermocellum* [42].

**Fig. 4:**
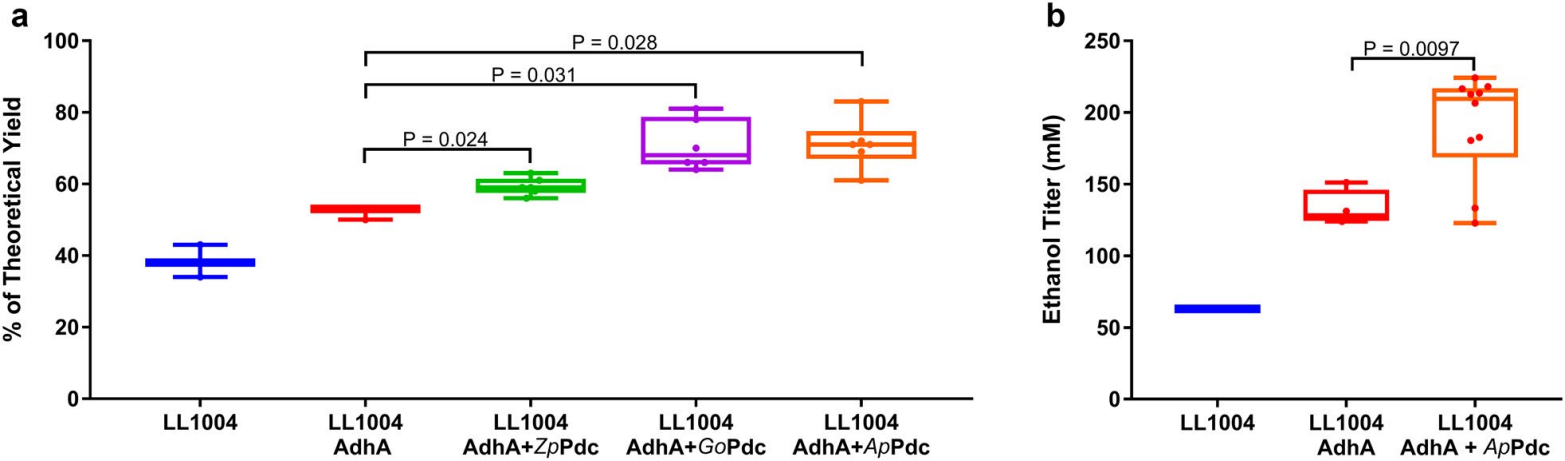
The ethanol production of *C. thermocellum* with different pyruvate decarboxylase genes and *T. saccharolyticum* alcohol dehydrogenase. **a** Two colonies were selected for each candidate *pdc* gene and evaluated by batch fermentation in MTC-5 medium with 30 g/L cellobiose. Three biological replicates were measured. **b** Ten colonies with AdhA and *Ap*Pdc were cultured in MTC-5 medium with 30 g/L cellobiose. Data for each colony is represented by a single point. All the *P* values were calculated based on the control strain LL1004 with only AdhA. 10 μg/mL thiamphenicol was added to maintain the plasmid. The box includes the 25th to 75th percentile of data; whiskers represent 1.5 times the interquartile range

The *adhA* and *pdc* genes were cloned into a plasmid. Protein expression was optimized by targeted modification of the RBS region using a web-based tool [43, 44]. A plasmid containing only the *adhA* gene was also constructed as a control. The new plasmids were used to transform the wild type strain of *C. thermocellum* (LL1004). Two colonies of each transformation were selected and then cultured in MTC-5 medium with 30 g/L cellobiose. The ethanol yields (percentage of the theoretical maximum yield of four moles of ethanol per mole of cellobiose consumed) of the colonies with *Zp*Pdc, *Go*Pdc and *Ap*Pdc are shown in Fig. 4a.

Among these *pdc* genes, the strains with *Ap*Pdc produced the highest titer. Although strains expressing the other two Pdc proteins also show similar yields, their titers were much lower compared to the strain expressing *Ap*PDC. To determine the colony-to-colony variation, eight more colonies with *Ap*Pdc were selected and cultured in MTC-5 medium with 30 g/L cellobiose (Fig. 4b; Additional file 3). Most of the colonies produced more than 200 mM ethanol, 54% more than the control strain (AdhA without Pdc). The strain expressing AdhA and *Ap*Pdc exerted an inhibitory effect on growth which reduced the growth rate by ~ 12% (Additional file 4). Colony LL1398 (expressing *Ap*PDC and AdhA) was the best ethanol producing strain, based on the 30 g/L cellobiose fermentation experiment, and was selected for further study.

### Confirmation of PDC activity in *C. thermocellum*

Transcription of the *pdc* gene was confirmed by qPCR (Additional file 5: Figure S1). Both the *adhA* and *pdc* genes showed high levels of expression (relative to the *recA* reference gene). However, we were unable to accurately measure PDC activity from cell extract of C*. thermocellum*. The background activity was quite high because *C. thermocellum* has a number of enzymes which could interfere with the PDC activity assay, including lactate dehydrogenase, pyruvate ferredoxin oxidoreductase and pyruvate formate lyase. To avoid this interference, we purified the *Ap*Pdc from *C. thermocellum* using a 6× His-tag. The purified enzyme had a specific activity of 0.28 ± 0.04 U/mg, which is about 100 times lower than the activity of the *Ap*Pdc cloned and purified from *E. coli* (31.07 ± 1.50 U/mg). One possible explanation for this difference is that in *C. thermocellum*, the protein was kept at 55 °C for a longer period of time (i.e. the time when the organism was growing, before the protein was harvested), compared to *E. coli*, which was grown at 37 °C. Another possible explanation is that Pdc acquires post-translational modifications in *E. coli* that it does not receive in *C. thermocellum*. Yet another possibility is that a large portion of the enzyme is degraded by native protease enzymes in *C. thermocellum*, but not in *E. coli*.

### Batch culture of the strain with *Ap*Pdc and AdhA

**Fig. 5:**
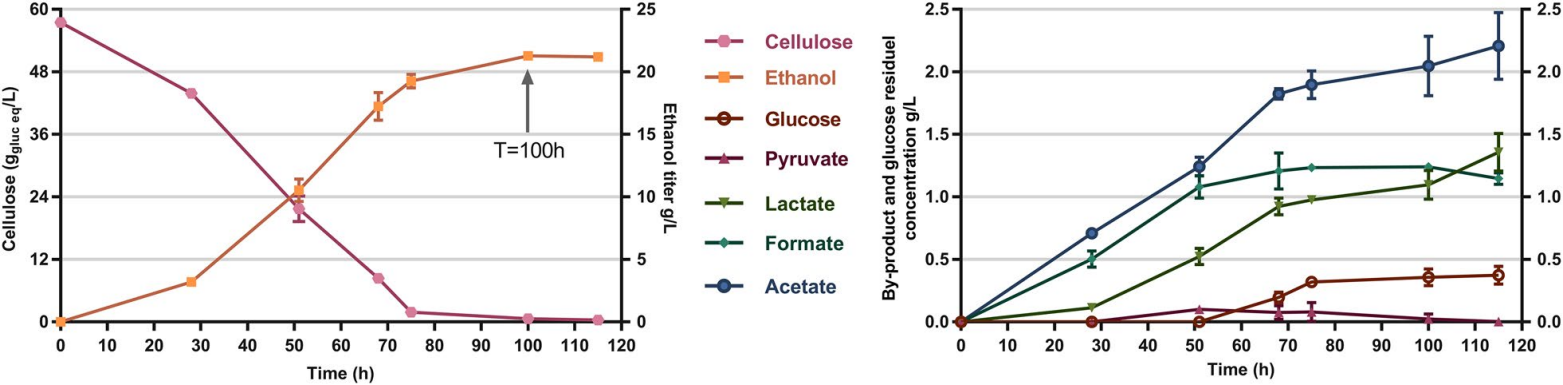
Cellulose consumption and product formation by the strain with AdhA and *Ap*PDC at 55 °C and pH 6.5 in a bioreactor. The strain was grown in pH-controlled bioreactors, with 60 g/L crystalline cellulose as the sole carbon source. Error bars on each data point represent one standard deviation (n = 3 fermentations)

In a bioreactor batch fermentation of strain LL1398 (expressing *Ap*PDC and AdhA) with 60 g/L cellulose (Avicel PH105), 95% of the substrate was consumed (Fig. 5; Additional file 6). The final ethanol titer was 21.3 ± 0.2 g/L and the yield was 0.35 g_EtOH_/g_Glu eq_, which is 70% of the maximum theoretical yield. The main by-products were lactate (1.1 ± 0.1 g/L), acetate (2.0 ± 0.2 g/L) and formate (1.2 ± 0.1 g/L).

The carbon recovery based on the T = 100 h sample was calculated and the total carbon recovery was 100.7% (Table 2). Besides biomass (8.0%), the main fermentation by-products were extracellular amino acids which accounted for 11.8% of the carbon. Glutamate (1.7 ± 0.3 g/L), valine (0.6 ± 0.2 g/L), arginine (0.5 ± 0.1 g/L) and alanine (0.4 ± 0.02 g/L) were the most abundant amino acids. In comparison to the amino acid production of wild type and some engineered *C. thermocellum* strains, the concentrations of amino acids of the glutamate family (glutamate and arginine) were much higher. In contrast, the concentrations of amino acids of the pyruvate family (alanine and valine) were much lower [10, 45]. The changes in amino acid concentration may be the result of *pdc* and *adhA* overexpression.

**Table 2:** Carbon balance from fermentation of 60 g/L cellulose (328 mM glucose equivalents)

Compound	mM	%C3 (pyruvate)^a^
Ethanol	460.9	70.3%
Ex amino acid carbon (pyruvate equivalents)^b^	77.3	11.8%
Biomass carbon	165.0	8.0%
Ex protein carbon^c^	55.2	2.8%
Acetate	31.4	4.8%
Lactate	13.4	1.9%
Ex sugar^d^	8.3	1.2%
Total		100.7%

^a^To facilitate comparison, carbon-containing compounds were expressed in terms of C3 equivalents as described previously [10]. For example, one C3 equivalent (i.e. pyruvate) is required to produce one ethanol

^b^Ex amino acid carbon; amount of carbon in extracellular free amino acids

^c^Ex protein carbon; amount of carbon in extracellular (secreted) protein

^d^Ex sugar; extracellular sugar, including all the soluble glucan and xylan

### Whole-genome sequencing

To identify secondary mutations, two colonies from strains expressing *Zp*Pdc, *Go*Pdc and *Ap*Pdc were sent for whole genome sequencing (Additional file 7). Fermentation profiles of these strains are shown in Additional file 8. No mutations were found in the *pdc* or *adhA* genes. Most of mutant genes have no clear function. The functions of mutant genes Clo1313_0217 (UDP-N-acetylglucosamine pyrophosphorylase), Clo1313_1397 (copper amine oxidase-like domain-containing protein) and Clo1313_2174 (RNA polymerase sigma-I factor) do not seem to be directly related to ethanol production. However, two other mutations have annotations related to protease activity. One is Clo1313_0988, which is annotated as a proteinase inhibitor. The mutation in the RBS region of Clo1313_0988 is unique to strain LL1398 (the highest ethanol producer). Based on the calculated RBS activity [43, 44], the mutation should result in increased translation of the Clo1313_0988 gene. If this proteinase inhibitor is targeting proteinases that target Pdc or AdhA, its overexpression might improve the life-time of these proteins. To test this hypothesis, we cloned the Clo1313_0988 gene into one of the plasmid expressing the *ApPdc* and *adhA* genes and transformed it into *C. thermocellum*. However, we did not observe a significant change in ethanol production.

The other is protease-related mutation is Clo1313_1489, which is annotated as a peptidase. The mutation in Clo1313_1489 is a transposon insertion that presumably leads to a loss of function, which could improve PDC activity if Clo1313_1489 is responsible for PDC degradation, however this hypothesis awaits experimental confirmation.

### Plasmid stability

In this work, *pdc* and *adhA* genes were expressed on a plasmid, however we observed that in some cases up to 45% of the cells had lost the plasmid at the end of the fermentation (CFU assay result, Additional file 9). One possible solution to overcome the problem of plasmid instability would be to integrate the *pdc* and *adhA* genes on the genome.

## Conclusion

In this work, the pyruvate decarboxylase from *A. pasteurianus* was heterologously expressed in *C. thermocellum*. With the help of an alcohol dehydrogenase from *T. saccharolyticum*, both the ethanol yield and titer were significantly increased. The final engineered strain, LL1398, produced ethanol from cellulose at 70% of the maximum theoretical yield and titer of 21.3 g/L. This technique could readily be applied to other thermophilic organisms.

## Methods

### Strain construction and molecular techniques

All the chemicals ordered were from Sigma-Aldrich (St. Louis, MO) or Fischer Scientific (Pittsburgh, PA) and were of molecular grade unless otherwise stated. Primers were ordered from Integrated DNA Technologies (Coralville, IA). Strains, plasmids, and primers can be found in Additional files 10, 11, 12. Transformation of plasmid DNA into to *C. thermocellum* was performed as previously described [46, 47]. Plasmid sequences are available from Genbank (see Accession Numbers in Additional file 11). Genetic modification was confirmed by PCR and whole genome sequencing.

### Media and growth

Strains were grown anaerobically at 55 °C for all experiments. For biochemical characterization and transformation, strains were grown in CTFUD medium with 5 g/L cellobiose in a COY (Ann Arbor, MI) anaerobic chamber (85% N_2_, 10% CO_2_, and 5% H_2_) [46]. For quantification of fermentation products, strains were grown in sealed serum bottles on 30 g/L cellobiose in 50 mL of defined MTC-5 medium with a N_2_ headspace. The medium recipe and bottle preparation has been described previously [10]. Bioreactor fermentations were carried out in 1.5-L (1-L working volume) Sartorius Biostat A-plus Sartorius Stedim (Sartorius Stedim, Bohemia, NY) bioreactors in modified MTC-5 medium without MOPS buffer, with the temperature maintained at 55 °C and stirred at 150 rpm. The pH was controlled at 6.5 with a Mettler-Toledo pH probe (Columbus, OH) by the addition of 8 N KOH. The vitamin supplementation solution contained pyridoxamine dihydrochloride 0.04 g/L, PABA 0.008 g/L, d-biotin 0.004 g/L, vitamin B-12 0.004 g/L. The vitamin supplementation solution was filter sterilized and added after autoclaving the bioreactor. The bioreactor was inoculated with 5% v/v transfer of a fresh seed culture grown on 5 g/L Avicel PH105 in MTC-5 (0.5% v/v). The headspace of the bioreactor was flushed with an anaerobic gas mixture (80% N_2_ and 20% CO_2_) prior to inoculation. Thiamphenicol (dissolved in dimethyl sulfoxide) was added to the medium to a final concentration of 15 µg/mL as a selective agent to maintain the plasmid.

For growth rate measurements, 200 µL cultures were grown in 96-well pre-sterilized polystyrene plates in an anaerobic chamber (85% N_2_, 10% CO_2_, and 5% H_2_). Absorbance measurements (OD_600_) were taken every 10 min for 36 h using a Biotek plate reader (BioTek Instruments Inc., Winooski, VT). Growth rates were determined based on the slope of log-transformed absorbance data. Growth rates were determined from the mean of five independent biological replicates.

### Heterologous protein expression in *E. coli*

Target genes were amplified by PCR with Q5 DNA polymerase (New England Biolabs, Ipswich, MA USA). Genomic DNA from each target organism was used as the template. The primers used for each gene are listed in Additional file 12. The target genes were inserted into plasmid pD861-CH (DNA2.0 Inc., Menlo Park, CA, USA) and tagged with a C-terminal His_6_ cassette. The vector was transformed into *E. coli* BL21(DE3).

Cells were grown aerobically in TB medium at 37 °C with a stirring speed of 225 rpm. When the OD_600_ reached 0.6, 4 mM rhamnose was added to induce the expression of the target gene. The cells were then grown aerobically for 4 h before harvesting by centrifugation. The cell pellets were washed with buffer (50 mM Tris–HCl, 0.5 mM DTT pH 7.5) and stored at − 80 °C.

### Preparation of cell extracts

*Escherichia coli* cells were prepared as described in “Confirmation of PDC activity in *C. thermocellum*”. *C. thermocellum* cells were harvested by centrifugation when the absorbance at 600 nm reached a value of 0.6.

The cell pellet was resuspended in lysis buffer (1 × BugBuster reagent (EMD Millipore, Darmstadt, Germany) with 0.2 mM dithiothreitol). The cells were lysed with Ready-Lyse lysozyme (Epicentre, Madison, WI, USA), and DNase I (New England Biolabs, Ipswich, MA, USA) was added to reduce the viscosity. After incubation for 30 min at room temperature, the resulting solution was centrifuged at 10,000×*g* for 5 min. The supernatant was used as cell extract for enzyme assays or purification.

### Protein purification

All purification steps were performed at room temperature as described previously [17]. His-tag affinity spin columns (His SpinTrap; GE Healthcare BioSciences, Pittsburgh, PA, USA) were used to purify the protein. The column was first equilibrated with binding buffer (50 mM sodium phosphate, 500 mM NaCl, 20 mM imidazole, pH 7.5). Cell extracts (in 50 mM sodium phosphate, 500 mM NaCl, 20 mM imidazole, pH 7.5) were applied to the column, and then the column was washed twice with wash buffer (50 mM sodium phosphate, 500 mM NaCl, 50 mM imidazole, 20% ethanol, pH 7.5). The His-tagged protein was eluted with elution buffer (50 mM sodium phosphate, 500 mM NaCl, 500 mM imidazole, pH 7.5).

For large volume purification, Ni Sepharose high performance affinity media (GE Healthcare BioSciences, Pittsburgh, PA, USA) and Glass Econo-Column^®^ Columns 2.5 × 10 cm (BioRad, Hercules, CA, USA) were used.

### Pyruvate decarboxylase assay

The pyruvate decarboxylase activity was measured using a coupled assay with yeast alcohol dehydrogenase [26]. The reaction mix contained: 50 mM pH 7.0 Tris–HCl buffer (The pH was adjusted at 25 °C), 2 mM MgCl_2_, 0.2 mM thiamine pyrophosphate, 0.3 mM NADH, 10 mM pyruvate and 1 U/mL of yeast alcohol dehydrogenase from Sigma (A3263). Pyruvate was used to start the reaction and the consumption of NADH was followed spectrophotometrically at 340 nm (molar extinction coefficient ε of NADH = 6.22 mM^−1^ cm^−1^). The protein concentration was determined using the Bradford protein reagent with bovine serum albumin as the standard (BioRad, Hercules, CA).

For the thermostability assay, the Pdc protein was mixed with the reaction mix without NADH and Adh and then incubated at various temperatures for 30 min. The pH of reaction mix was set to 7.0 at 25 °C. Then the incubated mix was chilled on ice for 15 min before the assay. Both NADH and Adh protein could be used to start the reaction. In cases where Adh protein was used to start the reaction, the rate of the reaction was determined after the reaction had reached steady state (i.e. the brief, transient initial rate was ignored).

### Analytical methods

Acetate, formate, ethanol, glucose, and cellobiose were determined by high pressure liquid chromatography (HPLC, Waters, Milford, MA) with refractive index detection using an Aminex HPX-87H column (Bio-Rad, Hercules, CA) with a 2.5 mM sulfuric acid solution as the mobile phase. Pellet nitrogen was determined using a Shimadzu TOC-VCPH total organic carbon analyzer with added total nitrogen unit (Shimadzu Scientific Instruments, Columbia, MD), calibrated using an acidified glycine standard [48]. 1 mL samples were centrifuged at 15,000×*g* for 10 min, the supernatant was discarded, and the pellet was rinsed twice using equal volumes of deionized purified water (MilliQ). Residual Avicel PH105 concentration was determined in units of glucose equivalents by quantitative saccharification as previously described [45]. Supernatant protein was determined with the Bradford assay (Thermo Scientific, Rockford, IL) with bovine serum albumin (BSA) (Thermo Scientific, Rockford, IL) as a standard. The concentrations of secreted amino acid were determined using an Aracus amino acid analyzer (membraPure, Berlin, Germany) as previously described [8]. Briefly, after separation using a T111 Li-cation exchange column, amino acids were derivatized with ninhydrin. Derivatized amino acids were detected at a wavelength of 570 nm, and concentrations were calculated by comparing peak areas to those of a standard amino acid mixture.

### Carbon balance calculations

Carbon balances were calculated as described previously [49]. The molar concentration of Avicel PH105 was calculated based on glucose monomers with a formula weight of 163 g/mole and 2 C3 equivalents per glucose. For glucose, extracellular sugar (non-glucose), and isobutanol, each mole was assumed to be equivalent to 2 C3 units. Ethanol, acetate, malate and lactate were assumed to be equivalent to 1 C3 unit. For amino acids, the number of C3 units was based on calculations from Stephanopoulos et al. 1998 [50]. Alanine (1 C3 equivalent) and valine (2 C3 equivalents) account for the majority of the amino acid carbon. For biomass, 1 mol of pellet carbon was assumed to be equivalent to 1/3 mol of C3 units and based upon measured pellet nitrogen values [48]. Extracellular protein was converted to C3 equivalents by assuming that extracellular protein is 45% carbon by mass.

### Quantitative PCR

Quantitative PCR was performed as previously described [13]. Cultures were harvested to mid-exponential phase (OD_600_ between 0.6 and 1.0) and then treated with RNA protect bacteria reagent (Qiagen Catalog Number 76506) per kit instructions. The primers used for qPCR are listed in Additional file 12: Table S3. Gene expression in all strains were normalized against *C. thermocellum recA* expression [52].

## Additional files

**Additional file 1.** The absolute specific activities of the purified eight pyruvate decarboxylases.

**Additional file 2.** The pyruvate decarboxylase specific activities from *Clostridium thermocellum* cell extract.

**Additional file 3.** Fermentation product and sugar residules of 10 *Clostridium thermocellum* colonies overexpressing AdhA and *Ap*PDC.

**Additional file 4.** Growth rate comparison.

**Additional file 5: Figure S1.** Quantitative PCR result of *Appdc* gene and *adhA* gene in *C. thermocellum*.

**Additional file 6.** Residual substrate and products concentration during fermentation.

**Additional file 7.** Sequencing data of selected strains.

**Additional file 8.** Fermentation data of sequenced strains.

**Additional file 9.** Assay of plasmid loss by CFU count.

**Additional file 10.** Strains used in this study.

**Additional file 11.** Plasmids used in this study.

**Additional file 12.** Primers and gblocks used in this study.

## Abbreviations

ADH: alcohol dehydrogenase
ALDH: aldehyde dehydrogenase
BSA: bovine serum albumin
CBP: consolidated bioprocessing
CFU: colony forming unite
MOPS: 3-morpholino-propane-1-sulfonic acid
PDC: pyruvate decarboxylase
PFOR: pyruvate ferredoxin oxidoreductase
FNOR: ferredoxin-NAD(P)^+^ oxidoreductase
Fdo: oxidized ferredoxin
FdR: reduced ferredoxin
SNV: single nucleotide variation
