# Supplementary material for: Enhanced ethanol formation by Clostridium thermocellum via pyruvate decarboxylase
Source: Microb Cell Fact. 2017 Oct 4;16:171. doi: 10.1186/s12934-017-0783-9 (PMC5628457; doi:10.1186/s12934-017-0783-9)
Supplement: Supplementary file 1 — Additional file 1. The absolute specific activities of the purified eight pyruvate decarboxylases. [file 12934_2017_783_MOESM1_ESM.docx]

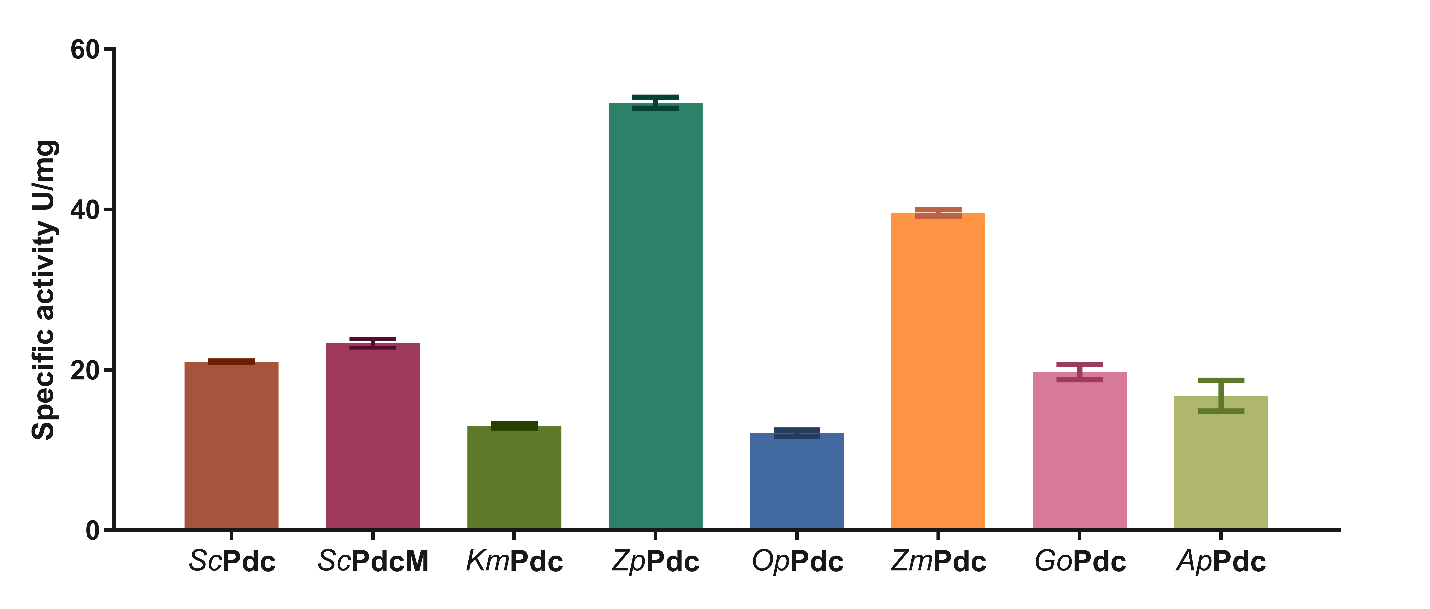


**Additional file 1: The absolute specific activities of the purified eight pyruvate decarboxylases.** Enzyme activities were measured at 30 °C. The data represents the average of three individual rounds of protein purification and assay.
