# Supplementary material for: Enhanced ethanol formation by Clostridium thermocellum via pyruvate decarboxylase
Source: Microb Cell Fact. 2017 Oct 4;16:171. doi: 10.1186/s12934-017-0783-9 (PMC5628457; doi:10.1186/s12934-017-0783-9)
Supplement: Supplementary file 2 — Additional file 2. The pyruvate decarboxylase specific activities from Clostridium thermocellum cell extract. [file 12934_2017_783_MOESM2_ESM.docx]

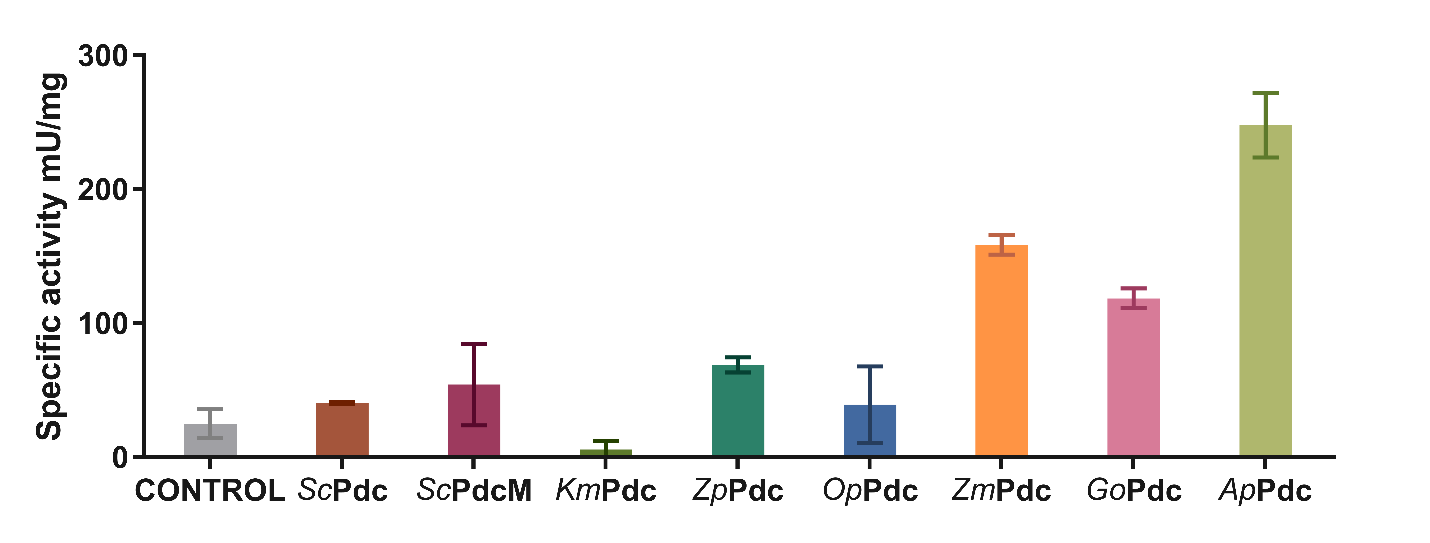


**Additional file 2:** **The pyruvate decarboxylase specific activities from *Clostridium thermocellum* cell extract.** To avoid the background activity, *pdc* genes were overexpressed in a mutant strain of *C. thermocellum* with lactate dehydrogenase deleted. The cell extract from that mutant was used as a control. Enzyme activities were measured at 30 °C. The data represents the average of three individual rounds of protein purification and assay. Error bar represent one standard deviation.
