# Supplementary figures and images for: Enhanced ethanol formation by Clostridium thermocellum via pyruvate decarboxylase
Source: Microb Cell Fact. 2017 Oct 4;16:171. doi: 10.1186/s12934-017-0783-9 (PMC5628457; doi:10.1186/s12934-017-0783-9)

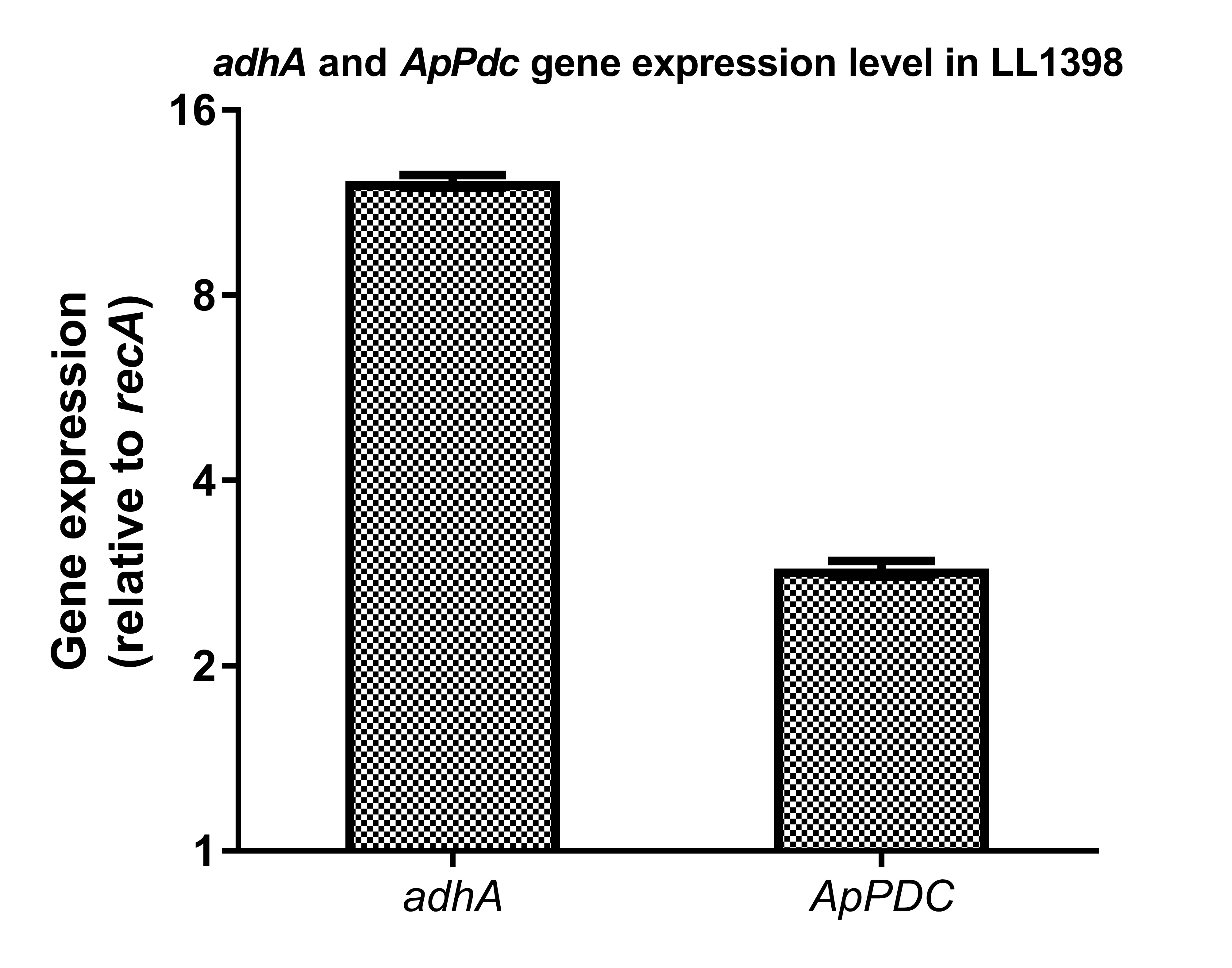

Supplement: Supplementary file 5 — Additional file 5: Figure S1. Quantitative PCR result of Appdc gene and adhA gene in C. thermocellum. [file 12934_2017_783_MOESM5_ESM.tif]
